# The ubiquitin specific protease USP34 promotes ubiquitin signaling at DNA double-strand breaks

**DOI:** 10.1093/nar/gkt622

**Published:** 2013-07-17

**Authors:** Shirley M. H. Sy, Jun Jiang, Wai Sum O, Yiqun Deng, Michael S. Y. Huen

**Affiliations:** ^1^Genome Stability Research Laboratory, The University of Hong Kong, Hong Kong, People’s Republic of China, ^2^Department of Anatomy, The University of Hong Kong, Hong Kong, People’s Republic of China, ^3^Center for Cancer Research, LKS Faculty of Medicine, The University of Hong Kong, Hong Kong, People’s Republic of China, ^4^Guangdong Provincial Key Laboratory of Protein Function and Regulation in Agricultural Organisms, College of Life Sciences, South China Agricultural University, Guangzhou, Guangdong 510642, China and ^5^State Key Laboratory of Brain and Cognitive Science, The University of Hong Kong, Hong Kong, People’s Republic of China

## Abstract

Ubiquitylation plays key roles in DNA damage signal transduction. The current model envisions that lysine63-linked ubiquitin chains, via the concerted action of E3 ubiquitin ligases RNF8-RNF168, are built at DNA double-strand breaks (DSBs) to effectively assemble DNA damage-repair factors for proper checkpoint control and DNA repair. We found that RNF168 is a short-lived protein that is stabilized by the deubiquitylating enzyme USP34 in response to DNA damage. In the absence of USP34, RNF168 is rapidly degraded, resulting in attenuated DSB-associated ubiquitylation, defective recruitment of BRCA1 and 53BP1 and compromised cell survival after ionizing radiation. We propose that USP34 promotes a feed-forward loop to enforce ubiquitin signaling at DSBs and highlight critical roles of ubiquitin dynamics in genome stability maintenance.

## INTRODUCTION

Protein ubiquitylation is an evolutionarily conserved post-translational modification system that operates under three common enzymatic steps, and it involves the concerted action of ubiquitin activating enzyme (E1), ubiquitin conjugating enzyme (E2) and ubiquitin ligase (E3). In addition to those that covalently attach to its substrates on one (mono-ubiquitylation) or multiple (multi-mono-ubiquitylation) sites, protein substrates can be modified by ubiquitin polymers linked via one of its seven lysines (lys6, lys11, lys27, lys29, lys33, lys48 or lys63) or its N-terminal methionine (M1) (1). While substrates bearing lys48-linked ubiquitin chains are generally targeted for proteasomal degradation, recent studies have highlighted use of other ubiquitin topologies as signaling intermediates that mediate protein–protein interactions (2,3). In this context, although it has become clear that ubiquitin chains of distinct topologies are endowed with capacities to differentially regulate the fate of its substrates, much of the molecular events that underlie the exquisite regulation of this highly versatile system remain unknown.

Like many other post-translational modification systems, ubiquitylation status of protein substrates is dynamically regulated by the opposing activities of ubiquitin ligases and deubiquitinases (DUBs) (4). Accumulating evidence indicate that the balance of protein ubiquitylation orchestrates many fundamental cellular processes, and that compromising its reversibility has profound effects in cell homeostasis and organismal survival. Considering that the human genome encodes >600 ubiquitin ligases, the predicted ∼80 active DUBs suggest that each DUB may counteract the action of multiple ubiquitin ligases. Thus, one would envisage additional layers of fine tuning, compatible with the broader roles of each DUB, to ensure substrate specificity and spatiotemporal activation.

DNA damage triggers a series of signaling events that rely heavily on usage of non-degradative ubiquitin modifications. The E3 ubiquitin ligases RNF8 and RNF168 have emerged as core intermediates in DNA damage signal transduction, and in concert with the E2 UBC13, synthesise lys63-linked ubiquitin chains on H2A-type histone molecules surrounding DNA double-strand breaks (DSBs) (5). These DSB-associated ubiquitin conjugates allow efficient local assembly of DNA damage-repair factors, including tumor suppressors BRCA1 and 53BP1. Failure to amplify the DNA damage signal results in attenuated cell cycle checkpoint function and impaired DNA repair (3,6,7). Apart from lys63-linked ubiquitin conjugates, DSBs are also demarcated by mono-ubiquitylated species of H2A and H2B histones (8–10). Interestingly, mono-ubiquitylated histones have been implicated in nuclear architecture regulation, governing chromatin access and transaction events that operate on this dynamic entity. Indeed, RNF8 and RNF168 promoted H2A mono-ubiquitylation to suppress transcription at sites distal to DSBs (10), and the RNF20/RNF40 heterodimer facilitated DNA repair processes by regulating histone H2B ubiquitylation status (8,9). Although exactly how these events are coupled remains obscure, it has become clear that the ubiquitin environment is more complex than anticipated, and that distinct ubiquitin conjugates may dictate specific cellular functions that together promote optimal cell survival in response to genotoxic stress.

Unlike mono-ubiquitylated histones and lys63-linked ubiquitin conjugates, role of ubiquitin chains of other linkages in DNA damage signaling is not clear (2). Early studies suggested that the E3 ubiquitin ligase BRCA1 synthesizes lys6-linked ubiquitin chains (11,12). However, despite its presence at DSBs (13,14), its bona fide substrate and its functional relevance remains undetermined. More recent work implicated lys48-linked ubiquitin chains in proper DNA damage signaling, where removal of lys48-linked ubiquitin conjugates from DSBs represents a pre-requisite of full emanation of DNA damage signals arising from DSBs (15,16).

In this study, we describe the identification of USP34, which plays a role at DSBs by stabilizing RNF168, a key ubiquitin conjugate-promoting factor. USP34 enforces RNF168 functions in assembling DNA damage checkpoint and repair proteins at DSBs, failure of which compromises cell survival in response to genotoxic stress.

## MATERIALS AND METHODS

### Cell cultures and transfection

The 293T and HeLa cells were cultured in Dulbecco’s modified Eagle’s medium with 10% fetal bovine serum at 37°C in 5% CO_2._ Cells were transfected with Lipofectamine 2000 or oligofectamine (Invitrogen) according to the manufacture’s protocol.

### Antibodies and siRNAs

Antibodies against γH2AX, 53BP1, BRCA1, EXPAND1/MUM1, CtIP, MDC1, RAP80, RAD18, RNF8 and RNF168 were previously described (17–21). Conjugated ubiquitin was detected by anti-FK2 (Upstate). Anti-Flag (M2) and anti-actin antibodies were from Sigma. Anti-p53 (DO1) antibodies were from Santa Cruz. Anti-USP34 antibodies were from Abnova (clone 2E2) and Bethyl (A300–824A). Anti-ubiquitin (P4D1–A11), anti-ubiquitylated H2A (E6C5), anti-lys48-linked ubiquitin and anti-CHK1 pS345 antibodies were from Millipore. Anti-KAP1 and KAP1-S824P antibodies were from Transduction Lab and Bethyl, respectively. Anti-RAD18 antibodies were from Abnova and Bethyl. BRCA1-, RAP80-, RAD18-, 53BP1-, RNF8- and RNF168-targeting small-interfering RNAs (siRNAs)- were described previously (18,19,21,22), and USP34 siRNAs were from Dharmacon.

## Co-immunoprecipitation and immunoblotting

Cells were harvested and lysed with NETN buffer [20 mM Tris–HCl (pH 8), 100 mM NaCl, 1 mM EDTA, 0.5% Nonidet P-40] 24 h post-transfection. Protein extracts were cleared by centrifugation at 13 000 rpm for 10 min at 4°C. Ten percent of extract was used to detect the expression of transfected proteins, whereas the remaining was incubated with streptavidin-conjugated beads for 4 h at 4°C with gentle agitation. Beads were washed twice and boiled in SDS sample buffer for 10 min. Eluted proteins were then fractionated by SDS–PAGE and electroblotted to Hybond-P membranes followed by incubation in 5% skim milk for 30 min. Membranes were incubated with primary antibodies at 4°C overnight and subsequently incubated with horseradish peroxidase (HRP)-conjugated secondary antibodies (Jackson Immunoresearch Laboratories) for 4 h. Signals on the membrane were visualized by enhanced chemiluminescence (Thermo Fisher Scientific).

### Cycloheximide chase experiment

To determine RNF168 protein stability, USP34 siRNA or control siRNA-transfected cells were seeded onto 60 mm dishes supplemented with 50 μg/ml of cycloheximide. Cells were harvested at indicated time points, and lysates were separated by SDS–PAGE to analyze protein expressions at indicated time points.

### Immunofluorescence staining

Cells were grown as monolayers on coverslips and exposed to ionizing radiation. Cells were washed with phosphate buffered saline and fixed with 3% paraformaldehyde for 15 min at room temperature. Subsequently, cells were permeabilized in 0.5% triton solution for 2 min at room temperature. To detect ubH2A foci, cells were pre-extracted with triton solution followed by fixation with paraformaldehyde. Foci were stained by sequential incubations of primary antibodies and secondary fluorophore-conjugated antibodies for 30 min, respectively. Nuclei were counterstained with 4′,6-diamidino-2-phenylindole (DAPI).

### Clonogenic survival assay

siRNA-transfected cells seeded onto 60 mm^2^ culture dishes were irradiated with 0, 1, 2 and 5 Gy ionizing radiation (IR). Cells were allowed to grow for 14 days at 37°C, and the numbers of colonies were counted using QuantityOne. Three independent experiments were performed each in triplicates.

## RESULTS

### USP34 promotes cellular responses to DNA damage

**Figure 1.**
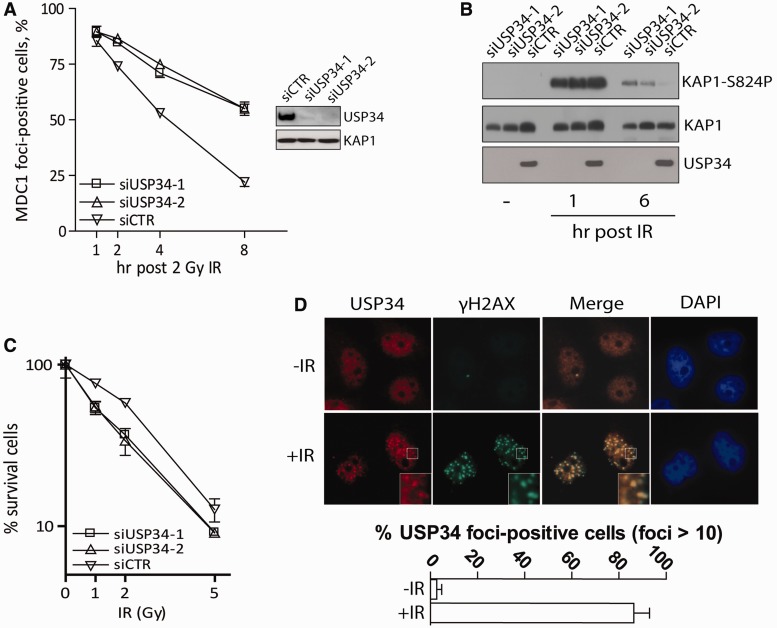
USP34 is a DNA damage-responsive protein. (**A**) HeLa cells transfected with USP34-targeting siRNAs (siUSP34-1 and siUSP34-2) or control siRNAs (siCTR) were seeded onto coverglasses before 2 Gy IR treatment. Cells were fixed after recovery and immunostained with anti-MDC1 antibodies to evaluate DNA repair kinetics. Western blotting experiments showed depletion of endogenous USP34 in cells treated with USP34-specific siRNAs. KAP1 expression was used as loading control. Experiments were repeated twice; (**B**) siRNA-treated cells were irradiated (10 Gy) and were subsequently lysed at indicated time points to evaluate level of phosphorylated KAP1 (KAP1-S824P); (**C**) HeLa cells transfected with indicated siRNAs were seeded onto 60 mm^2^ dishes, irradiated and allowed to recover for 14 days before the number of colonies were counted. Results represent data from two independent experiments each performed in triplicates; (**D**) HeLa cells were either untreated or treated with 10 Gy IR. Cells were processed for immunofluorescence experiments using indicated antibodies, and the percentage of USP34 foci-positive cells (of a total of 100 nuclei) before and after IR is plotted.

To explore roles of DUBs in DNA damage signaling, we studied the ubiquitin specific protease USP34, which was recently reported to stabilise Axin by removing ubiquitin chains, which would otherwise target Axin for proteasomal degradation (23). USP34, identified as a component that resided within the NBS1 protein complex (data not shown), encodes a predicted 3546 amino acid protein. Interestingly, the DUB was previously identified as a phosphoprotein in proteomic analyses for ATM/ATR substrates (24,25), with implicated roles in checkpoint control and DNA repair (24). Consistently, siRNA-mediated depletion of USP34 compromised DNA repair, as reflected by persistent MDC1 and γH2AX foci after low doses of IR challenge (Figure 1A and Supplementary Figure S1A and B) and by reduced rates of gene conversion events (Supplementary Figure S1C). Further, USP34-depleted cells displayed aberrant DNA damage signaling, as illustrated by immunoblotting for phosphorylated KAP1 (Figure 1B) and phosphorylated CHK1 (Supplementary Figure S1D), and were hyper-sensitized to IR in the clonogenic survival assay (Figure 1C). Together, we concluded that USP34 is important for cellular responses to DNA damage.

### USP34 localizes at DNA damage sites

**Figure 2.**
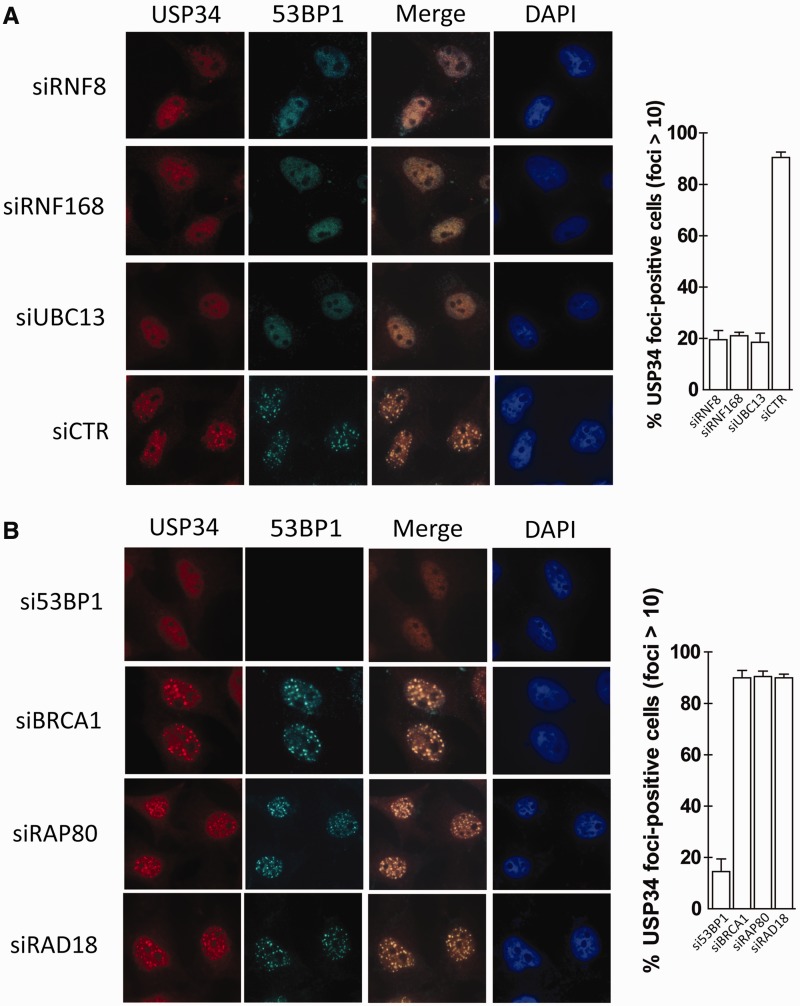
USP34 accumulation at DSBs requires the canonical DNA damage signaling pathway. (**A** and **B**) HeLa cells pre-treated with RNF8-, RNF168-, UBC13-, 53BP1-, BRCA1-, RAP80- RAD18-targeting or control siRNAs (siCTR) were seeded onto coverslips, irradiated (10 Gy) and processed 4 h after for indirect immunofluorescence staining using indicated antibodies. Percentage of USP34 foci-positive cells (of a total of 100 nuclei) is plotted for each siRNA treatment.

A common attribute of DNA damage-repair factors is their ability to localize at DSBs. We found that USP34 relocalized into punctuate structures that overlapped extensively with the DNA damage marker γH2AX (Figure 1D), suggesting direct roles of USP34 at DSBs. As chemical inhibition of ATM using KU55933 inhibited USP34 IRIF (Supplementary Figure S2), we examined whether DSB association of USP34 may require an intact H2AX-dependent DNA damage signaling pathway. Indeed, focal accumulation of USP34 at DNA breaks required the core ubiquitin enyzmes RNF8 and RNF168, and their cognate E2 UBC13 (Figure 2A). Surprisingly, we found that USP34 IRIF also required 53BP1, but not RAP80-BRCA1 or RAD18 (Figure 2B and Supplementary Figure S3). Consistent with pivotal roles of ubiquitin conjugates for USP34 IRIF, ectopically expressed RNF169, a recently identified antagonist of the RNF8/RNF168-mediated DNA damage ubiquitin signaling pathway (26–28), dislodged USP34 from DSBs (Supplementary Figure S4).

### USP34 stablizes RNF168 E3 ubiquitin ligase

**Figure 3.**
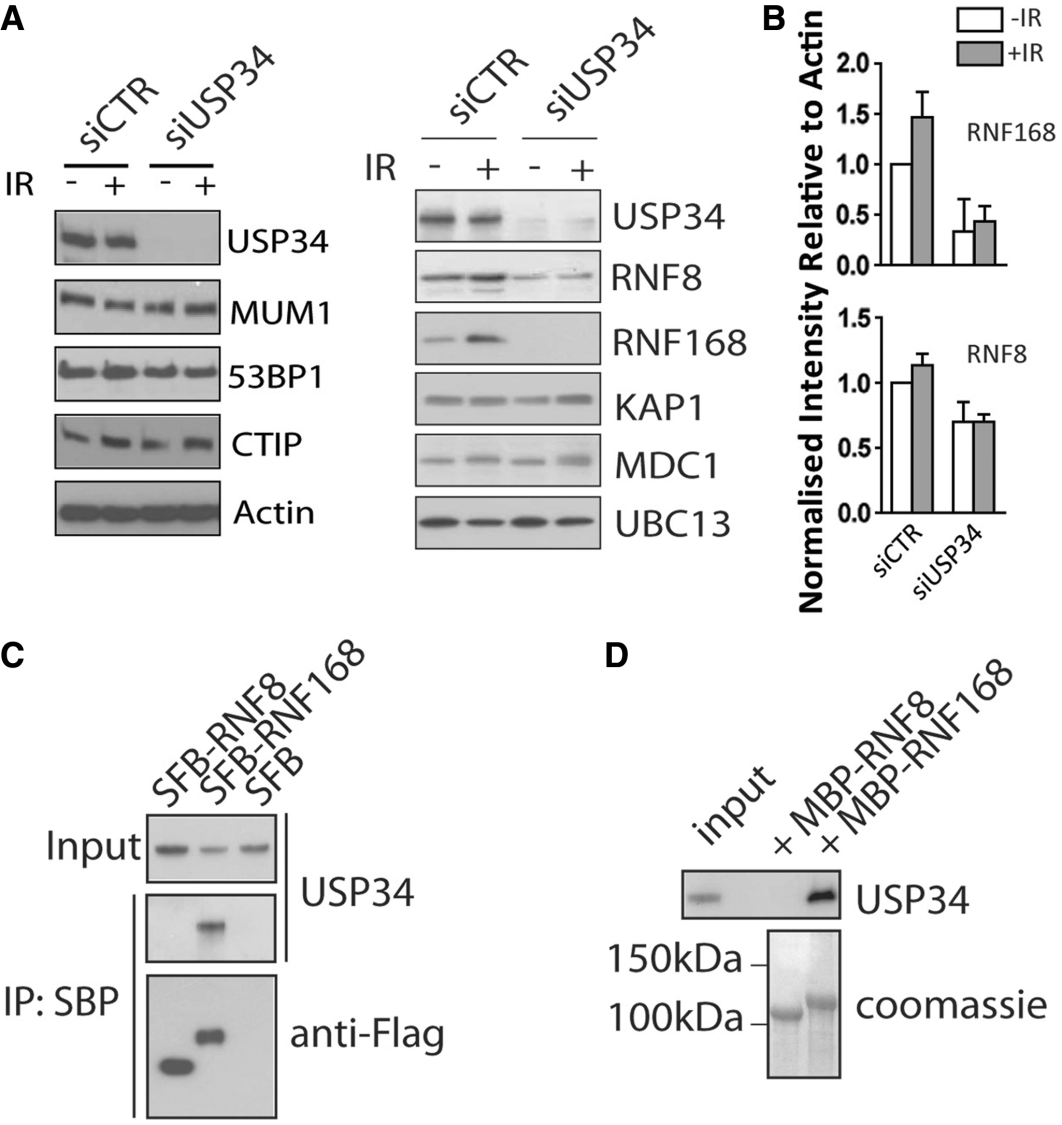
USP34 promotes RNF168 protein stability. (**A**) USP34-depleted or control cells were irradiated (10 Gy) or left untreated. Whole-cell extracts were prepared 4 h post-IR, and western blotting experiments were performed using indicated antibodies; (**B**) Relative expression of RNF168 (upper panel) and RNF8 (lower panel) normalized to Actin in (A) is shown. (**C**) 293 T cells were transfected with Streptavidin-binding peptide-Flag (SFB)-tagged RNF8, RNF168 or empty vector. Twenty-four hours post-transfection, cells were lysed in NETN, cleared by centrifugation, and streptavidin beads were added to immunoprecipitate protein complexes. (**D**) Amylose beads conjugated with bacterially expressed and purified MBP-RNF8 or MBP-RNF168 were incubated with 293 T cell lysate in NETN buffer for 4 h with gentle agitation at 4°C. Thereafter protein complexes were washed twice with NETN, separated by SDS–PAGE and analyzed by immunoblotting for USP34.

**Figure 4.**
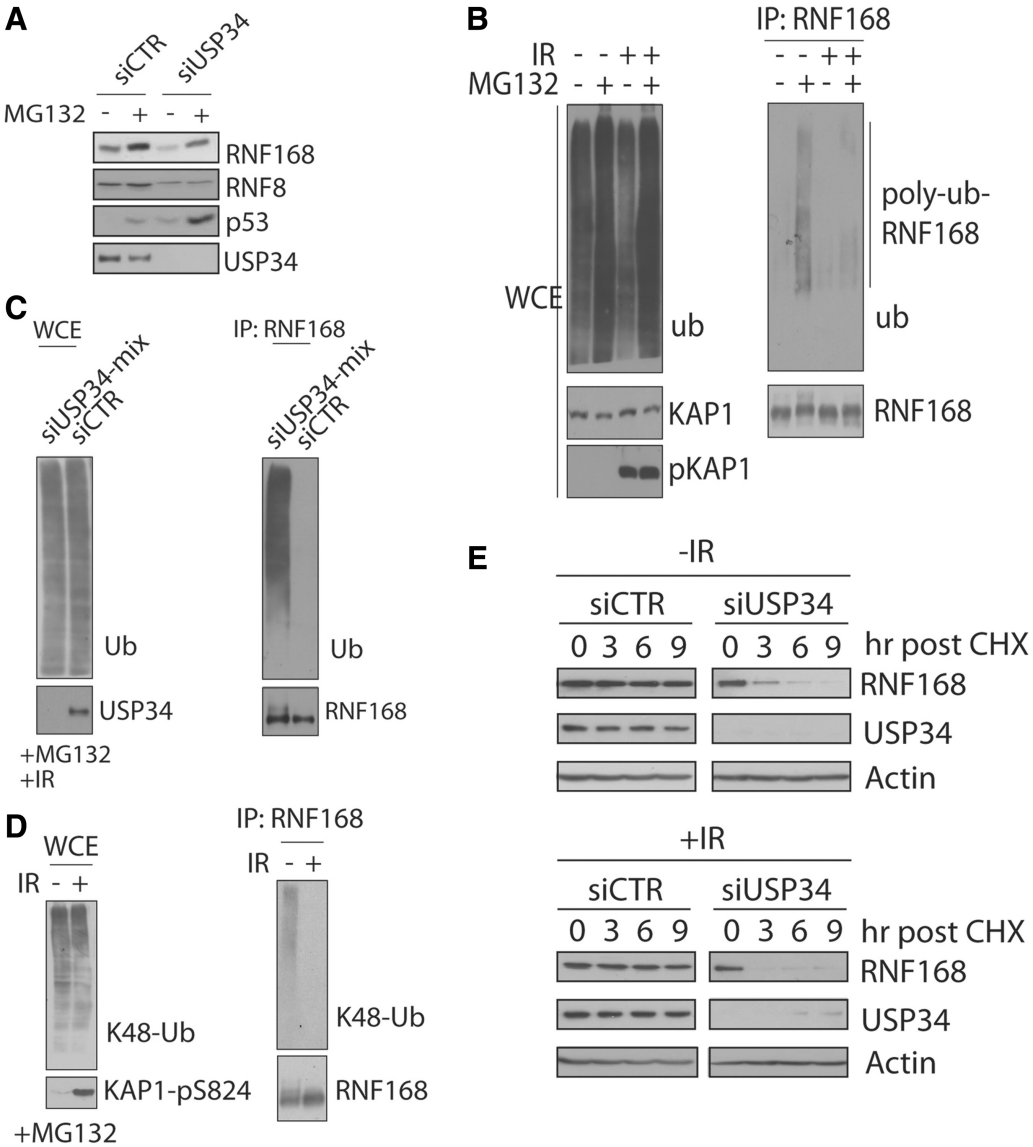
USP34 counteracts IR-induced RNF168 ubiquitylation. (**A**) HeLa cells transfected with indicated siRNAs were either untreated or pre-incubated with 10 µM MG132 for 4 h. Thereafter, whole-cell extracts were prepared to analyze expression level of indicated proteins, (**B–D**) HeLa cells incubated with Dulbecco’s modified Eagle’s medium supplemented with 10 µM MG132 were irradiated (10 Gy) or untreated. Cells were lysed 4 h post IR and RNF168 proteins were immunoprecipitated under denaturing conditions. Western blotting was performed to analyze ubiquitylation of RNF168 using indicated antibodies; (**E**) siRNA-transfected HeLa cells were treated with cycloheximide. Whole-cell extracts were prepared at indicated time points after addition of cycloheximide to evaluate expression of RNF168 in the presence or absence of USP34. Actin expression was used as loading control.

Given that USP34 is an enzymatically active DUB that stabilized Axin1 from proteaosomal degradation (23,29), we examined whether USP34 is required for maintaining stability of protein components in the canonical H2AX-dependent DNA damage signaling pathway. Amongst these components, expression level of RNF168 (and RNF8, albeit to a less extent) was markedly reduced in USP34-depleted cells (Figure 3A and B). As USP34 interacted with RNF168, but not RNF8 (Figure 3C and D), we focused our attention to dissect the functional interaction of USP34 and RNF168, although we are fully aware of the possibility that RNF8 may also transiently associate with USP34 and may represent a bona fide target of USP34. Interestingly, RNF168 is stabilized on DNA damage (Figure 3A and B). In line with role of proteasome in regulating RNF168 turnover, incubation of proteasomal inhibitor MG132 stabilized RNF168, an effect which was more pronounced in USP34-depleted cells (Figure 4A and Supplementary Figure S5A). These data imply that RNF168 abundance may be subjected to stringent regulation by the opposing effects of the deubiquitylating enzyme USP34 and the proteasome machinery. To corroborate this idea, we first examined RNF168 ubiquitylation status in irradiated and control cells. We immunopreipitated RNF168 under denaturing conditions and found that ubiquitylated species of RNF168 was markedly reduced after IR treatment (Figure 4B and Supplementary Figure S5B), a phenomena that was reversed in cells depleted of USP34 (Figure 4C). A similar observation was seen when we immunoblotted immunoprecipitated RNF168 proteins with antibodies specific for lys48-ubiquitin linkages (Figure 4D), suggesting that USP34 may deubiquitylate and stablize RNF168. Indeed, cycloheximide chase analysis confirmed higher rate of RNF168 turnover in the absence of USP34, especially after IR treatment (Figure 4E and Supplementary Figure S5C). Finally, to test whether USP34 may stabilize RNF168 at sites of DNA damage, we depleted 53BP1, which inhibited USP34 IRIF formation, and assessed RNF168 expression by western blotting experiments. Interestingly, we found that the IR-induced stabilization of RNF168 was abrogated in 53BP1-knockdown cells (Supplementary Figure S6), suggesting that USP34 plays an active role in stabilizing RNF168 at DSBs.

### USP34 promotes DNA damage-induced histone ubiquitylation

**Figure 5.**
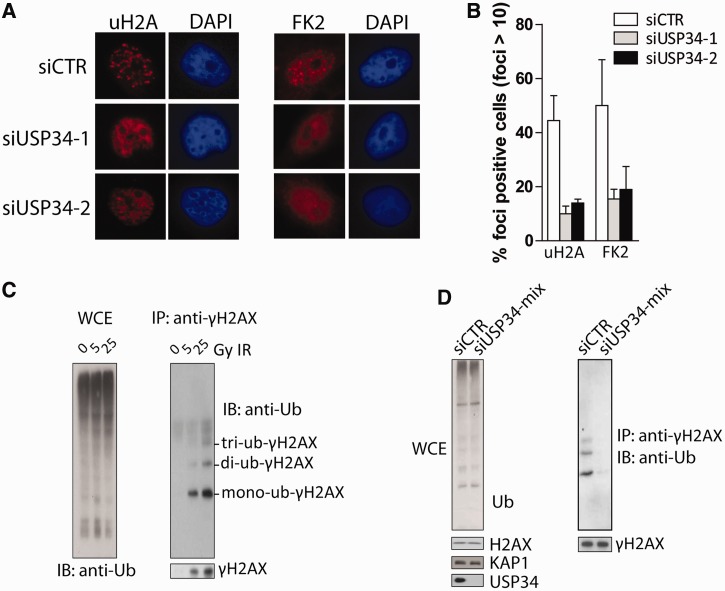
USP34 promotes DNA break-associated histone ubiquitylation. (**A** and **B**) HeLa cells were transfected with two independent USP34-targeting or control siRNAs. Cells were irradiated (3 Gy), allowed to recover for 1 h before processing for immunostaining experiments to examine focal accumulation of ubiquitylated H2A (uH2A) and conjugated ubiquitin (FK2). Percentage of uH2A and FK2 foci-positive cells (of a total of 100 nuclei) is plotted (B). (**C** and **D**) Ubiquitylation of γH2AX was examined 4 h after IR treatment (10 Gy). HeLa cells subjected to IR were lysed, sonicated and γH2AX was immunoprecipitated under denaturing condition. Western blotting experiments were performed using indicated antibodies.

DNA damage triggers RNF8/RNF168-dependent ubiquitylation of H2A-type histone molecules at DSBs (17,30–34). Given the putative role of USP34 in stabilizing RNF168, we tested whether USP34 may facilitate DSB-associated ubiquitylation events. Indeed, USP34 depletion attenuated formation of IR-induced ubiquitin conjugates at DSBs, as determined by immunostaining with low titration anti-ubiquitylated-H2A and anti-FK2 antibodies (Figure 5A and B). Furthermore, role of USP34 in facilitating RNF168 functions at DSBs is further illustrated biochemically by immunoblotting for ubiquitylated species of γH2AX histone molecules (Figure 5C). We concluded that USP34 promotes RNF168-dependent ubiquitylation events at DSBs.

### USP34 is required for assembly of DNA damage repair factors at DNA breaks

**Figure 6.**
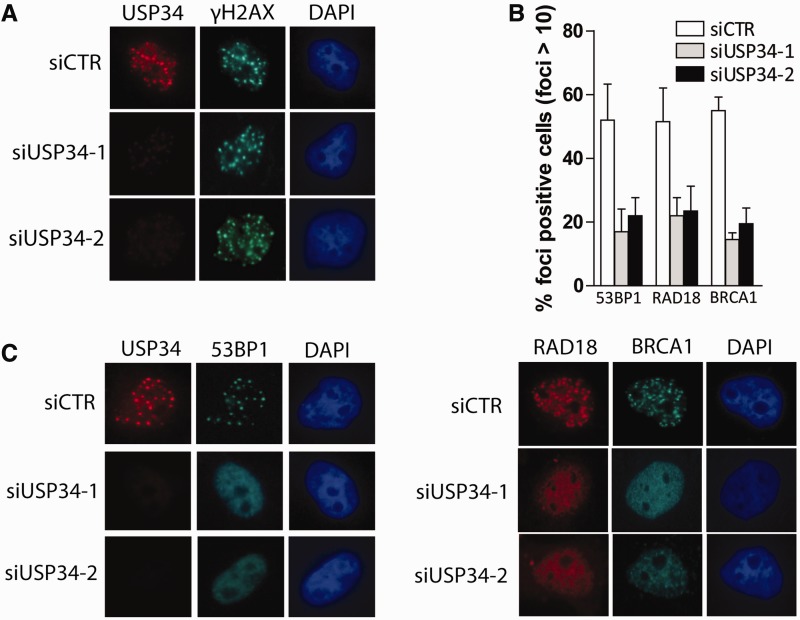
USP34 facilitates assembly of DNA damage repair proteins. (**A–C**) USP34-depleted (siUSP34-1 and siUSP34-2) or control cells (siCTR) were irradiated (3 Gy), allowed to recover for 1 h before processing for immunostaining experiments were performed to evaluate focal accumulation of USP34 (A), 53BP1, RAD18 and BRCA1 (C). Percentage of cells with >10 foci (of a total of 100 nuclei) were scored as positive and quantification data is plotted (B).

RNF168 works in concert with RNF8 at DSBs to amplify ubiquitin chains via lys63-linkages. Cells depleted of RNF168, and those derived from RIDDLE patients (RNF168 mutant), display defects in the accumulation of various DNA damage checkpoint and mediator proteins, including BRCA1, 53BP1 and RAD18 at DNA damage sites (18,33–35). In line with perturbed ubiquitin signaling at DSBs, USP34-depletion led to defective accrual of BRCA1, 53BP1 and RAD18 to DNA breaks (Figure 6A–C). Thus, USP34 encodes a DNA damage-responsive DUB that promotes RNF168 functions at DSBs.

## DISCUSSION

Studies from the past decade have illuminated the critically important roles of regulatory ubiquitylation in cellular responses to DNA damage. Accordingly, the ubiquitin-modified landscape at the damaged chromatin has emerged as focal points where DNA damage signals are transmitted and amplified through a series of signaling events, failure of which compromises DNA repair and cell survival. Importantly, despite the rapid progress in understanding how lys63-linked ubiquitin conjugates allow DNA damage signal propagation, potential role of lys48-linked ubiquitin chains at DSBs, perhaps hampered by its transient nature, has remained obscure. In this study, we have uncovered USP34 as a DSB-associated deubiquitylating enzyme that enforces DNA damage-induced ubiquitin signaling. By stabilizing RNF168, USP34 plays instrumental roles in the productive assembly of DNA damage checkpoint and mediator proteins at DSBs. The identification of USP34 thus provides an opportunity to further delineate how the proteasome may play an active role to limit DNA damage signaling.

Chromatin surrounding DSBs are decorated by a growing list of ubiquitin conjugates, including mono-ubiquitylated H2A/H2B histones (8–10), and lys63-linked ubiquitin modified H2A/X histones (17,30,33,34,36). Although these conjugates may serve distinct purposes during DNA damage-repair processes, the need to orchestrate these different outcomes requires multiple levels of regulation and fine-tuning. Indeed, the importance of achieving homeostatic balance of the dynamic ubiquitin environment is highlighted by the emergence of regulatory factors that innervate the ubiquitin-dependent DNA damage signaling cascade at the RNF8-RNF168 level (15,16,26–28,37–46). In particular, the ubiquitin-selective segregase VCP/p97 was recently found to promote DNA damage signaling by dismantling DSB-associated lys48-linked ubiquitin conjugates (15,16). Although one may be tempted to speculate that p97 may also act on ubiquitylated species of RNF168, the observation that P97 is not detectably required for lys63-linked ubiquitin chain formation at DSBs argues against this possibility and suggests that USP34 may operate in parallel pathways for proper cellular responses to genotoxic stress. Further experiments will be needed to consolidate this working model.

DNA damage resulted in substantial reduction of lys48 poly-ubiquitylated forms of RNF168, which coincided with the accumulation of RNF168 proteins. These results suggested that RNF168 may become stabilized on DSB docking. As USP34 accumulation at DNA breaks required the core ubiquitin signaling intermediates RNF8 and RNF168, we speculated that proteasome targeting of RNF168 may be counteracted by USP34 at DSBs. Indeed, not only does IR-induced stabilization of RNF168 require USP34 and its recruiting factor 53BP1, RNF168 protein half-life was much shorter in the absence of USP34. In addition, we found that ectopically expressed RNF168 was sufficient in restoring DSB-associated ubqiuitylation in USP34-depleted cells (Supplementary Figure S7). Based on these lines of evidence, we favor the idea that USP34, being an active DUB, may enzymatically trim away ubiquitin chains on DSB-associated RNF168 proteins, which may otherwise become degraded. Although this offers an attractive and highly tunable mechanism to stablize RNF168 proteins at DSBs, at this moment, we cannot exclude the possibility that USP34 may also stabilize RNF168 via direct protein–protein interactions, largely because the size of USP34 has precluded us from reconstituting USP34-depleted cells with its catalytic mutant allele. Further experimentations will be needed to differentiate these two possibilities.

During the preparation of this manuscript, Lukas and colleagues reported two HECT-domain containing E3 ligases that negatively regulate the abundance of RNF168 (47). The authors proposed that restricted accumulation of RNF168 at DSBs prevents excessive spreading of ubiquitin conjugates to undamaged chromosomal loci. Together with our study, these data strongly implicate RNF168 as a rate-limiting component in the ubiquitin-dependent DNA damage signaling pathway, and that positive and negative regulators have evolved to tightly modulate this important propagating step of ubiquitin signaling that emanates from DSBs. Moreover, given that depletion of USP34 resulted in reduction of both RNF8 and RNF168 proteins, it is possible that, apart from regulating RNF168, USP34 may act on other DSB-associated substrate. We are currently exploring this possibility.

Using affinity purification approaches, USP34 was recently identified as an Axin-interacting DUB that promoted Wnt/beta-catenin signaling (23). Notably, USP34 operated within the cell nucleus to stabilize the nuclear pool of Axin. It will be interesting to examine whether distinct pools of USP34 are designated for Wnt/beta-catenin and DNA damage signal transduction, or whether USP34 may serve as a bridging molecule that functionally links beta-catenin signaling with that of DNA damage responses. In summary, our study identifies USP34 as an integral component important for the functional interplay between lys48- and lys63-linked ubiquitin species at DSBs and highlights the dynamics of protein ubiquitylation in proper cellular responses to genotoxic stress.

## SUPPLEMENTARY DATA

Supplementary Data are available at NAR Online.

Supplementary Data
