# Supplementary material for: The ubiquitin specific protease USP34 promotes ubiquitin signaling at DNA double-strand breaks
Source: Nucleic Acids Res. 2013 Jul 17;41(18):8572–80. doi: 10.1093/nar/gkt622 (PMC3794584; doi:10.1093/nar/gkt622)
Supplement: Supplementary Data [file supp_41_18_8572__index.html]

The ubiquitin specific protease USP34 promotes ubiquitin signaling at DNA double-strand breaks — The ubiquitin specific protease USP34 promotes ubiquitin signaling at DNA double-strand breaks — Supplementary Data 

# The ubiquitin specific protease USP34 promotes ubiquitin signaling at DNA double-strand breaks

## 

files

**Files in this Data Supplement:**

- Supplementary Data - pdf file
