# Supplementary material for: The ubiquitin specific protease USP34 promotes ubiquitin signaling at DNA double-strand breaks
Source: Nucleic Acids Res. 2013 Jul 17;41(18):8572–80. doi: 10.1093/nar/gkt622 (PMC3794584; doi:10.1093/nar/gkt622)

Supplemental Figure Caption

Supplemental Figure 1. Role of USP34 in DNA Damage-Repair

A & **B**) siRNA-treated HeLa cells were left un-irradiated (no IR) or irradiated (3 Gy), fixed at indicated time points and were subsequently processed for indirect immunofluorescence experiments using anti-γH2AX antibodies. Percentage of γH2AX-positive cells (out of a total of 100 nuclei) is plotted (**B**). Experiment was repeated twice, **C**) DR-U2OS cells pre-treated with indicated siRNAs were transfected with expression construct encoding the ISceI endonuclease. 48 hr post-transfection the percentage of GFP positive cells was analysed by flow cytometry. PALB2, a homology-directed DNA repair protein, was used as positive control, **D**) HeLa cells treated with indicated siRNAs were irradiated with increasing doses of IR (0,1,5,10 Gy), lysed 1 hr post IR treatment and Western blotting experiments were performed using indicated antibodies.

Supplemental Figure 2. Chemical inhibition of ATM inhibited USP34 IRIF formation

HeLa cells grown on overslips were incubated with DMSO or ATM inhibitor KU55933 (10 μM) for 4 hr before IR treatment. Cells were processed for immunofluorescence studies using indicated antibodies. Nuclei was counterstained with DAPI.

Supplemental Figure 3. Effective siRNA-mediated knockdown of DSB repair proteins

HeLa cells were transfected twice at 24 hr intervals with indicated siRNAs. 48 hr post-transfection cells were harvested and whole cell extracts were prepared for Western blotting experiments using indicated antibodies.

Supplemental Figure 4. RNF169 inhibited USP34 IRIF formation

U2OS cells ectopically expressing Flag-tagged RNF169 were irradiated and processed for immunofluorescence studies using indicated antibodies. Cell nuclei were counterstained with DAPI.

Supplemental Figure 5. Quantitative analyses of RNF168 proteins

A-C) Relative expression of RNF168 proteins (**A** & **C**) or its ubiquitylated species (**B**) was determined using ImageJ software. Values represent mean$\pm$S.E.M. from at least two independent experiments.

Supplemental Figure 6. Role of 53BP1 in IR-induced stabilization of RNF168 proteins

HeLa cells pre-incubated with indicated siRNAs were irradiated (10 Gy) or left untreated. 4 hr post IR cells were lysed and whole cell extracts were separated by

SDS-PAGE. Expression of RNF168 was determined by Western blotting.

Supplemental Figure 7. Ectopically expressed RNF168 promoted DSB-ubiquitylation in USP34-depleted cells

U2OS cells pre-treated with USP34-targeting siRNAs were transfected with Flag-RNF18 expression constructs. 24 hr post-transfection cells were irradiated (3 Gy, 1 hr) and processed for immunofluorescence studies using indicated antibodies. Efficient knockdown of USP34 was validated by Western blotting using anti-USP34 antibodies.

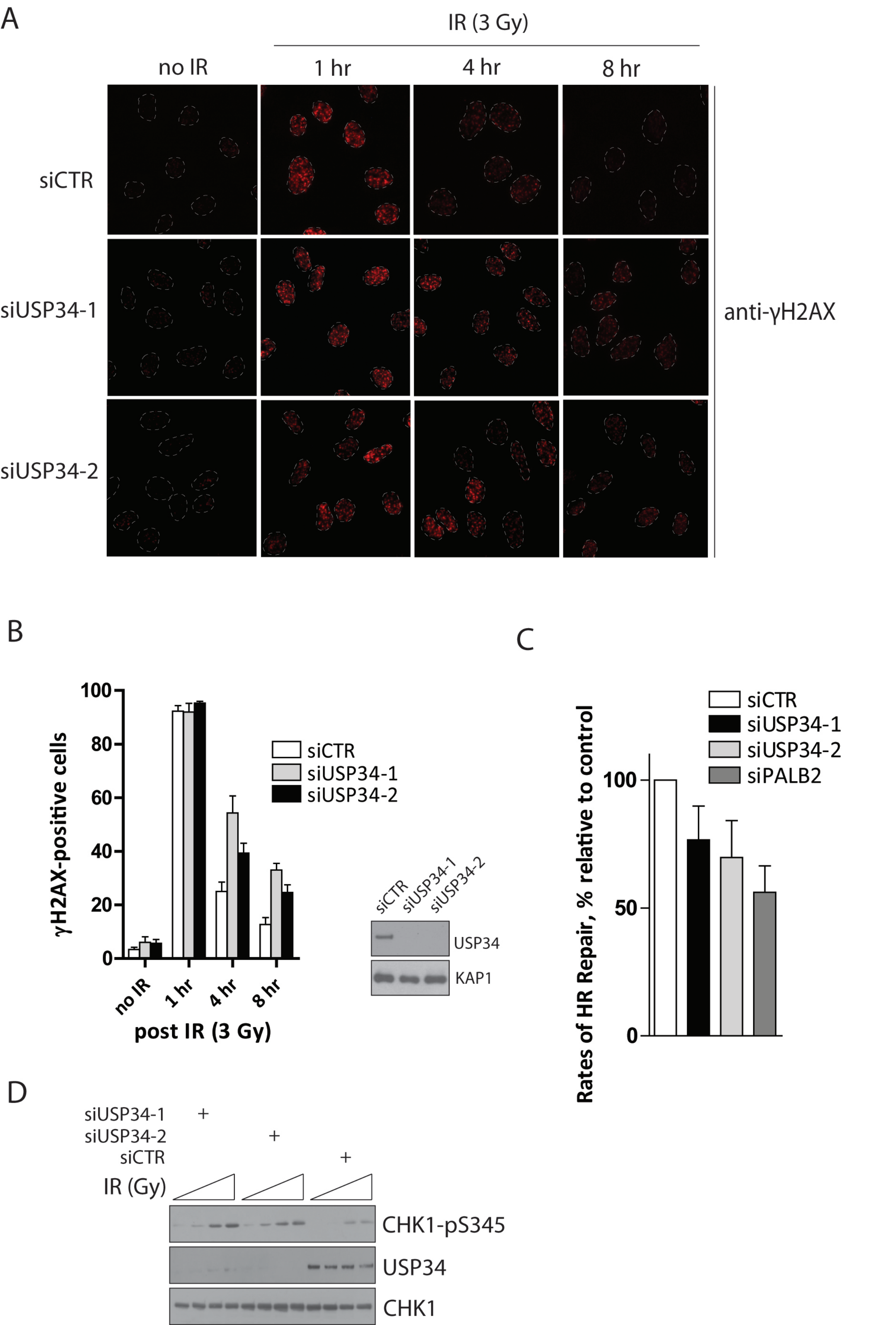
A
IR (3 Gy)
no IR
1 hr
4 hr
8 hr
siCTR
siUSP34-1
siUSP34-2
anti-γH2AX
B
γH2AX-positive cells
100
80
60
40
20
0
siCTR
siUSP34-1
siUSP34-2
no IR
1 hr
4 hr
8 hr
post IR (3 Gy)
siCTR
siUSP34-1
siUSP34-2
USP34
KAP1
C
siCTR
siUSP34-1
siUSP34-2
siPALB2
Rates of HR Repair, % relative to control
100
50
0
D
siUSP34-1 +
siUSP34-2 +
siCTR +
IR (Gy)
CHK1-pS345
USP34
CHK1

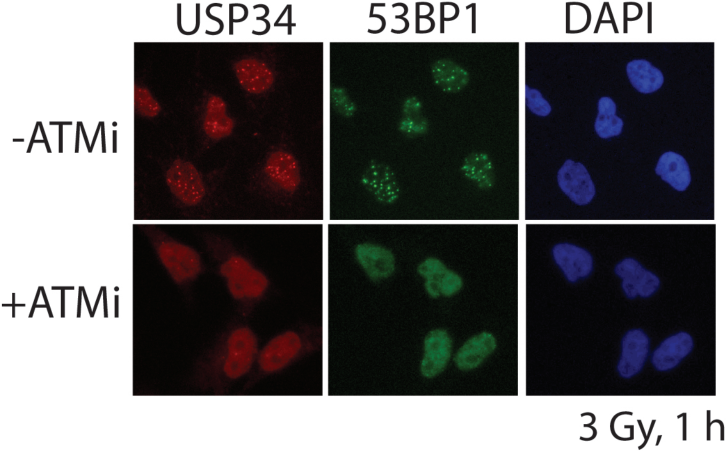
USP34
53BP1
DAPI
-ATMi
+ATMi
3 Gy, 1 hr

Sy_Supp Fig 3

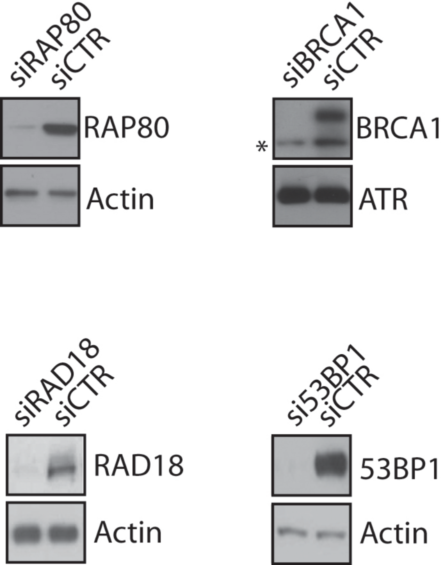
siRAP80
siCTR
RAP80
Actin
siBRCA1
siCTR
*
BRCA1
ATR
siRAD18
siCTR
RAD18
Actin
si53BP1
siCTR
53BP1
Actin

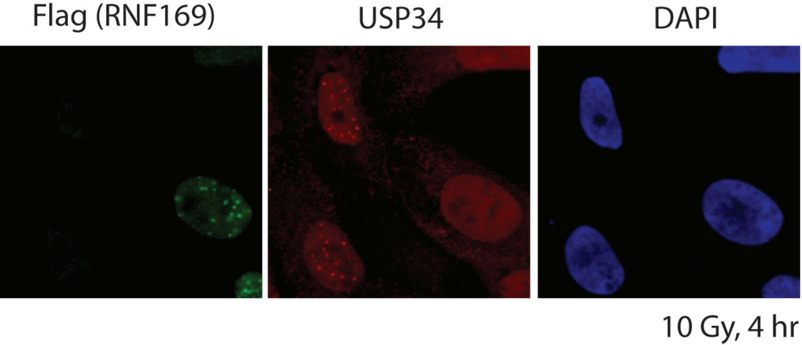
Flag (RNF169)
USP34
DAPI
10 Gy, 4 hr

A

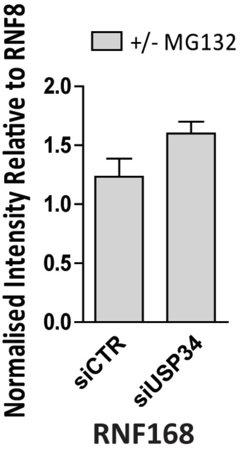
+/- MG132
Normalised Intensity Relative to RNF8
2.0
1.5
1.0
0.5
0.0
siCTR
siUSP34
RNF168

B

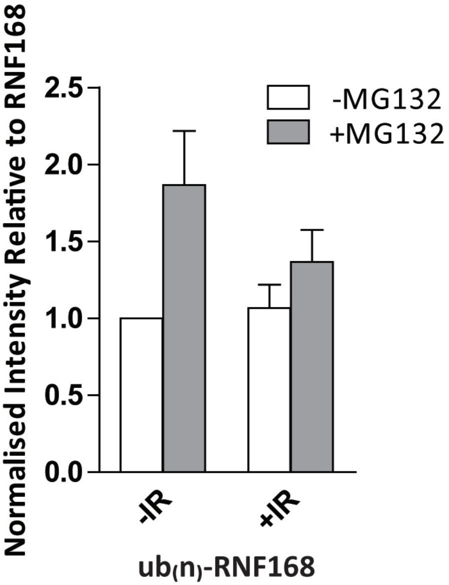
Normalised Intensity Relative to RNF168
2.5
2.0
1.5
1.0
0.5
0.0
-MG132
+MG132
-IR
+IR
ub(n)-RNF168

C

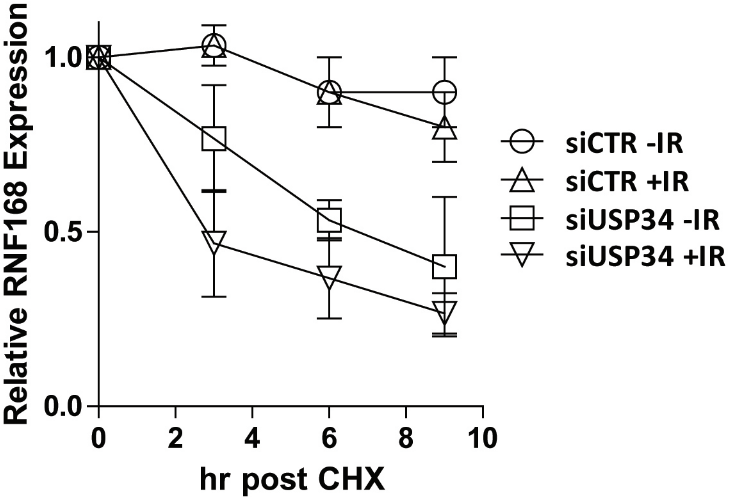
Relative RNF168 Expression
1.0
0.5
0.0
0
2
4
6
8
10
hr post CHX
siCTR -IR
siCTR +IR
siUSP34 -IR
siUSP34 +IR

Sy_Supp Fig 6

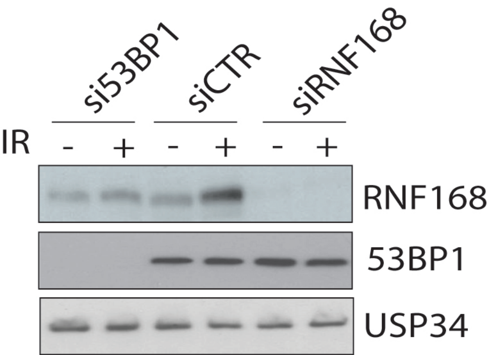

Sy_Supp Fig 7

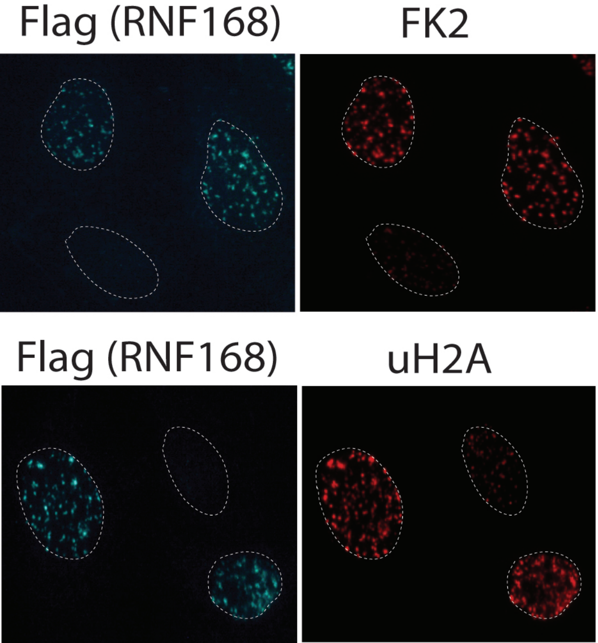

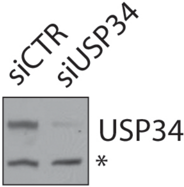

Supplement: Supplementary Data [file supp_gkt622_nar-03338-d-2012-File008.pdf]
